# Engineering and Modulating Functional Cyanobacterial CO_2_-Fixing Organelles

**DOI:** 10.3389/fpls.2018.00739

**Published:** 2018-06-05

**Authors:** Yi Fang, Fang Huang, Matthew Faulkner, Qiuyao Jiang, Gregory F. Dykes, Mengru Yang, Lu-Ning Liu

**Affiliations:** Institute of Integrative Biology, University of Liverpool, Liverpool, United Kingdom

**Keywords:** bacterial microcompartment, carboxysome, carbon fixation, cyanobacteria, encapsulation, Rubisco, self-assembly, synthetic engineering

## Abstract

Bacterial microcompartments (BMCs) are proteinaceous organelles widespread among bacterial phyla and provide a means for compartmentalizing specific metabolic pathways. They sequester catalytic enzymes from the cytoplasm, using an icosahedral proteinaceous shell with selective permeability to metabolic molecules and substrates, to enhance metabolic efficiency. Carboxysomes were the first BMCs discovered and their unprecedented capacity of CO_2_ fixation allows cyanobacteria to make a significant contribution to global carbon fixation. There is an increasing interest in utilizing synthetic biology to construct synthetic carboxysomes in new hosts, i.e., higher plants, to enhance carbon fixation and productivity. Here, we report the construction of a synthetic operon of the β-carboxysome from the cyanobacterium *Synechococcus elongatus* PCC7942 to generate functional β-carboxysome-like structures in *Escherichia coli*. The protein expression, structure, assembly, and activity of synthetic β-carboxysomes were characterized in depth using confocal, electron and atomic force microscopy, proteomics, immunoblot analysis, and enzymatic assays. Furthermore, we examined the *in vivo* interchangeability of β-carboxysome building blocks with other BMC components. To our knowledge, this is the first production of functional β-carboxysome-like structures in heterologous organisms. It provides important information for the engineering of fully functional carboxysomes and CO_2_-fixing modules in higher plants. The study strengthens our synthetic biology toolbox for generating BMC-based organelles with tunable activities and new scaffolding biomaterials for metabolic improvement and molecule delivery.

## Introduction

Subcellular compartmentalization is a hallmark of eukaryotic cells. It allows cells to perform and confine various chemical reactions in space and time and provide a means for eliminating metabolic crosstalk and enhancing the efficiency of compartmentalized metabolic pathways (Giessen and Silver, 2016; Hammer and Avalos, 2017). Compartmentalization also occurs in the prokaryotic cytoplasm and membranes, such as photosynthetic membranes (Liu, 2016). A particular example is bacterial microcompartments (BMCs), which are distributed in at least 23 different bacterial phyla, sequestering enzymes within the specialized niches from the cytoplasm (Axen et al., 2014; Chowdhury et al., 2014; Kerfeld and Erbilgin, 2015). All BMCs are made entirely of proteins, including interior enzymes that catalyze sequential reactions and an encapsulating shell. The architecture of the BMC shell resembles an icosahedral viral capsid, with facets composed of hexameric and trimeric proteins and vertices capped by pentameric proteins (Kerfeld et al., 2005; Tanaka et al., 2008; Klein et al., 2009; Sutter et al., 2017). These multiple protein paralogs have specific permeability for the passage of metabolites. Three types of BMCs have been extensively studied: the carboxysomes for CO_2_ fixation, the PDU BMCs for 1,2-propanediol utilization, and the EUT BMCs for ethanolamine utilization (Bobik et al., 2015; Kerfeld and Erbilgin, 2015).

The first BMCs identified were the carboxysomes, the central machinery for CO_2_ fixation in cyanobacteria (Shively et al., 1973). Composed of thousands of protein subunits, carboxysomes encapsulate the CO_2_-fixation enzyme ribulose-1,5-bisphosphate carboxylase/oxygenase (Rubisco) and carbonic anhydrase in a protein-based shell. Compartmentalization of these enzymes and the selective permeability of the shell that allows for the diffusion of HCO_3_^-^ and diminishes CO_2_ leakage ensure a high CO_2_ concentration near Rubisco (Cai et al., 2009), thereby favoring the higher carboxylation rates of Rubisco within carboxysomes and enhanced carbon fixation. According to the Rubisco phylogeny, protein composition and assembly, carboxysomes can be classified as α-carboxysomes (possessing Form 1A Rubisco) and β-carboxysomes (containing plant-like Form 1B Rubisco) (Rae et al., 2013). It has been deduced that α-carboxysomes and β-carboxysomes embrace distinct assembly pathways. The α-carboxysome shell assembles concomitantly with aggregation of Rubisco (Iancu et al., 2010) or even without Rubisco packing (Baker et al., 1998; Menon et al., 2008), whereas *de novo* assembly of β-carboxysomes follows the “inside out” mode: the β-carboxysome shell begins assembly after Rubisco aggregation (Chen et al., 2012; Cameron et al., 2013).

The CO_2_-fixing organelles in the model freshwater cyanobacterium *Synechococcus elongatus* PCC7942 (Syn7942) are β-carboxysomes, which have specific spatial location and regulation in cyanobacterial cells (Savage et al., 2010; Sun et al., 2016). The β-carboxysome shell in Syn7942 is composed of the hexameric proteins CcmK2-4 constructing shell facets (Kerfeld et al., 2005), CcmO (Rae et al., 2012), and the CcmL pentamers located at the vertices (Tanaka et al., 2008). Rubisco enzymes form a densely packed paracrystalline array in the β-carboxysome lumen (Faulkner et al., 2017). The packing of Rubisco is mediated by the 35 kDa truncated version of CcmM (CcmM35) (Long et al., 2010, 2011). The longer form of CcmM, Ccm58, contains an N-terminal domain with homology to γ-carbonic anhydrase and a C-terminal domain comprising three small subunit-like domains (SSUs) with homology to RbcS, the small subunit of Rubisco (Peña et al., 2010). CcmM interacts with the carboxysomal β-carbonic anhydrase (CcaA), Rubisco, and CcmN that acts as a bridge between CcmM and the shell (Long et al., 2007, 2010; Kinney et al., 2012). Though structurally resembling icosahedral virus capsids, the β-carboxysome from Syn7942 possesses a soft mechanical fingerprint (Faulkner et al., 2017).

The self-assembly, modularity, and metabolic enhancement of BMCs make these bacterial organelles an ideal engineering objective (Frank et al., 2013). BMC shell proteins have been shown to self-assemble to form flat sheets or tubular structures (Pang et al., 2014; Noël et al., 2016; Sutter et al., 2016), and empty shells (Lassila et al., 2014; Cai et al., 2015; Held et al., 2016; Sutter et al., 2017). Expression of *ccmK1*, *ccmK2*, *ccmL*, and *ccmO* from the cyanobacterium *Halothece* sp. PCC 7418 in *Escherichia coli* could generate the synthetic β-carboxysome shells, ∼25 nm in diameter (Cai et al., 2016). Likewise, empty BMC shells with the diameter of ∼40 nm were obtained by expressing a synthetic operon of *Haliangium ochraceum* BMC shell genes (Lassila et al., 2014; Sutter et al., 2017). The empty BMC shells, without encapsulated enzymes, are notably smaller than the native BMCs. Furthermore, previous studies have demonstrated the possibilities of engineering entire PDU BMCs from *Citrobacter freundii* and EUT BMCs from *Salmonella enterica* in *E. coli* (Parsons et al., 2008, 2010; Choudhary et al., 2012). Expressing the α-carboxysome operon from a chemoautotroph *Halothiobacillus neapolitanus* has also led to the production of recombinant carboxysome-like structures with CO_2_ fixation activity in *E. coli* (Bonacci et al., 2012) and in a Gram-positive bacterium *Corynebacterium glutamicum* (Baumgart et al., 2017). Apart from engineering BMCs in bacterial expression systems, efforts have been made to express β-carboxysome components in the chloroplasts of the model plant tobacco *Nicotiana benthamiana*. It was illustrated that transient expression of β-carboxysome proteins CcmK2, CcmM, CcmL, CcmO, and CcmN could lead to the formation of carboxysome-like circular structures (Lin et al., 2014a). Moreover, Rubisco enzymes with a high carboxylation rate could be produced in tobacco chloroplasts by importing Syn7942 Rubisco-CcmM35 complexes to replace endogenous plant Rubisco (Lin et al., 2014b; Occhialini et al., 2016). Despite the recent advancement of carboxysome engineering, until now, heterologous production of entire functional β-carboxysome structures in any non-native hosts has not been reported, to our knowledge.

In this study, we generated a synthetic operon consisting of 12 β-carboxysome genes from Syn7942 and expressed the construct in *E. coli* to produce synthetic β-carboxysome-like structures with CO_2_ fixation capacity. We further purified the synthetic β-carboxysomes and elucidated their structure, activity and interchangeability. This study empowers our toolbox for engineering functional BMC structures in heterologous organisms and emphasizes the necessity of optimizing the expression of carboxysome modules. It represents a step toward modulating BMC structures and functions using synthetic biology, to produce new nanobioreactors and biomaterials with expanded biotechnological applications.

## Results

### Expression of Syn7942 β-Carboxysomes in *Escherichia coli*

**FIGURE 1:**
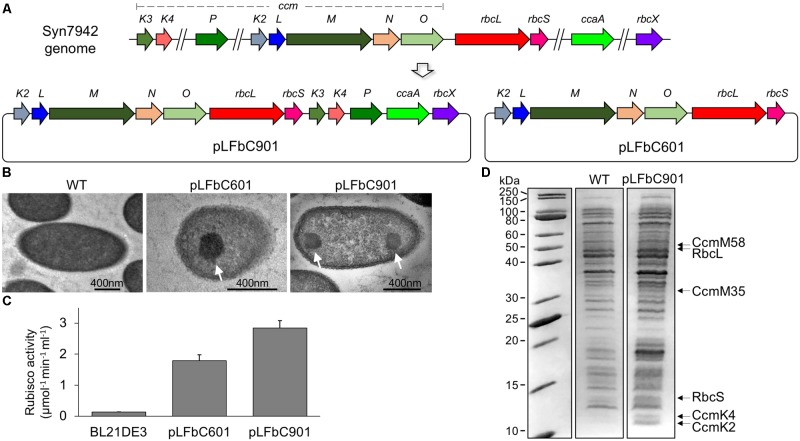
Synthetic operon design and heterologous expression of synthetic β-carboxysomes in *Escherichia coli*. **(A)** Schematic representation of the molecular organization of the natural β-carboxysome operons spread in five loci within the Syn7942 genome and the synthetic β-carboxysome operons (pLFbC901 and pLFbC601) inserted in the *E. coli* expression vector pETM11. Locus tags are indicated. **(B)** Thin-section TEM images of *E. coli* WT and cells expressing β-carboxysome proteins using pLFbC601 and pLFbC901 plasmids. Arrows indicate β-carboxysome-like structures with polyhedral shapes observed in pLFbC601 and pLFbC901 *E. coli* cells. **(C)** *In vivo* carbon fixation assays of *E. coli* WT, pLFbC901, and pLFbC601 cells, indicating the CO_2_-fixing activity of synthetic β-carboxysomes. A relatively higher CO_2_-fixing activity was determined in *E. coli* pLFbC901 cells compared with that of *E. coli* pLFbC601 cells. **(D)** SDS-PAGE of the total cell extracts of the recombinant *E. coli* pLFbC901 and the *E. coli* WT cells. Putative β-carboxysome proteins (RbcL, RbcS, CcmM, CcmK2, CcmK4) were identified based on their molecular weights. The presence of these β-carboxysome protein blocks was further confirmed by proteomic analysis (**Supplementary Table 2**). The gel image was spliced based on **Supplementary Figure 1**.

In contrast to the genes of α-carboxysomes that are mainly clustered together into a single operon, β-carboxysome genes typically appear in multiple dispersed gene clusters throughout the genome (Rae et al., 2013). In Syn7942, the twelve genes encoding β-carboxysome proteins are distributed in five different chromosomal loci and the major carboxysome genes are located in one locus containing the *ccmKLMNO* and *rbcLS* operons (**Figure 1A**). We generated a synthetic operon, by assembling all the twelve β-carboxysomal genes, to express β-carboxysome proteins in *E. coli* (**Supplementary Table 1**). Additionally, the *ccmKLMNO* and *rbcLS* operons were assembled into a single synthetic operon, for producing β-carboxysome structures with a reduced number of genes to be expressed. These two operons, containing the native promoters of individual carboxysome genes, were then inserted into an *E. coli* expression vector pETM11 under a T7 promoter, to generate the pLFbC901 and pLFbC601 constructs in the host *E. coli* BL21DE3 cells (**Figure 1A**).

Expression of β-carboxysome components using pLFbC901 and pLFbC601 was induced by IPTG. The formation of β-carboxysome structures was verified by thin-section electron microscopy. The carboxysome-like structures (∼200 nm in diameter) with a high internal protein density were observed in both recombinant *E. coli* cells harboring pLFbC901 and pLFbC601 but are invisible in the WT *E. coli* cells without carboxysome-expressing vectors (**Figure 1B**). Rubisco assays were performed to determine the *in vivo* carbon fixation activities of recombinant *E. coli* cells. Compared with WT cells, both pLFbC601 and pLFbC901 exhibit high carbon fixation activities, suggesting the functioning of recombinant carboxysome-like structures (**Figure 1C**). In addition, the recombinant *E. coli* cells containing pLFbC901 present a higher carbon fixation activity than the pLFbC601 cells, revealing the importance of CcaA, CcmK3, CcmK4, CcmP, and RbcX in maintaining the carboxysome structure and function. Considering the higher carbon fixation activity, we mainly focused on the pLFbC901 expression in the following study.

Expression of β-carboxysome protein components in the recombinant *E. coli* pLFbC901 was characterized by SDS-PAGE. **Figure 1D** illustrates that although the overall expression levels of β-carboxysome proteins are low, some β-carboxysome proteins are identifiable from the total cell extracts (see also **Supplementary Figure 1**). Proteomic analysis of the cell extracts further confirmed the presence of five β-carboxysome components, including the shell proteins (CcmK2, CcmK4), shell-associated proteins (CcmM), and internal proteins (RbcL, RbcS) (**Supplementary Table 2**). Some of the β-carboxysome proteins, namely CcmL, CcmO, CcmK3, CcaA, CcmN, CcmP, and RbcX, were not detectable. These downstream genes might be translated inefficiently, due to their distant locations to the inducible T7 promotor. Interestingly, the recent study illustrated that CcmN, CcmP, and RbcX were also not detectable in isolated β-carboxysomes from Syn7942 (Faulkner et al., 2017).

**FIGURE 2:**
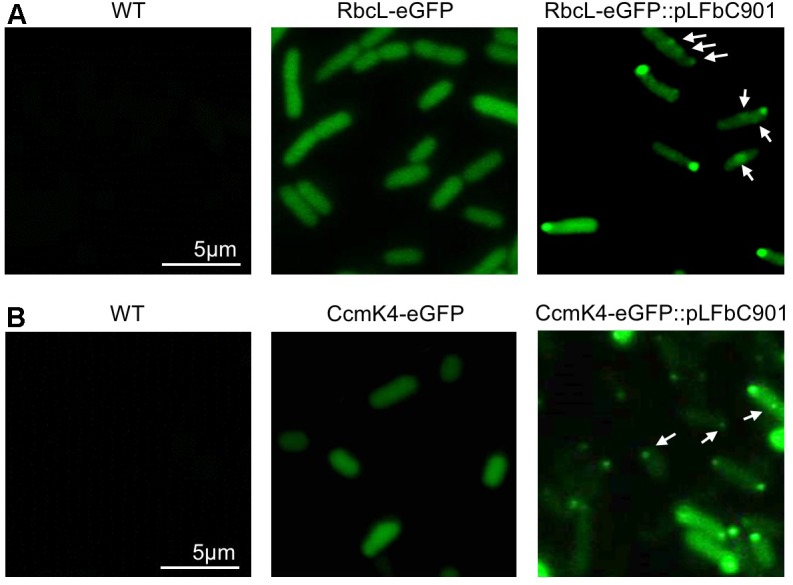
Fluorescence microscopy images of *E. coli* WT and recombinant cells expressing β-carboxysome components. **(A)** *E. coli* cells expressing RbcL-eGFP-RbcS in the absence or presence of the pLFbC901 vector. The *E. coli* WT cells were treated with the same conditions as the control. **(B)** *E. coli* cells expressing CcmK4-eGFP in the absence or presence of the pLFbC901 vector. The appearance of eGFP fluorescent puncta (arrows) indicates the assembly of β-carboxysome proteins in *E. coli*.

To evaluate the assembly of β-carboxysomal proteins in *E. coli*, we generated another two plasmids. One (RbcL-eGFP-RbcS) contains the *rbcLS* operon with the enhanced green fluorescence protein (eGFP) gene fused at the 3^′^ end of *rbcL*, which encodes the interior protein RbcL (Rubisco large chain) and the other (CcmK4-eGFP) comprises the gene of the shell protein CcmK4 fused with *egfp* at the 3^′^ end. The two plasmids were transformed into *E. coli* strains, respectively, and were expressed in the absence or presence of pLFbC901 (**Figure 2**). *E. coli* WT cells were treated in the same conditions as the control (**Figure 2**, left). Without the expression of pLFbC901, the expressed RbcL-eGFP-RbcS and CcmK4-eGFP are evenly distributed in the cytoplasm, and no detectable protein aggregation was visualized under this induction condition (**Figure 2**, middle). Co-expression of pLFbC901 with RbcL-eGFP-RbcS or CcmK4-eGFP induces the formation of protein assemblies (**Figure 2**, right), exhibiting strong fluorescence spots preferably at the cell polar and relative faint fluorescence spots in the cytoplasm (**Figure 2**, right, arrows). The cytoplasmic foci observed resemble the β-carboxysome structures observed in Syn7942 (Savage et al., 2010; Cameron et al., 2013; Sun et al., 2016), indicative of the formation of β-carboxysome structures; whereas the polar assemblies could be the inclusion bodies consisting of RbcL or CcmK4, as has been discerned in the heterologous expression of PDU BMCs and α-carboxysomes (Parsons et al., 2010; Bonacci et al., 2012).

### Purification and Characterization of Synthetic β-Carboxysomes

**FIGURE 3:**
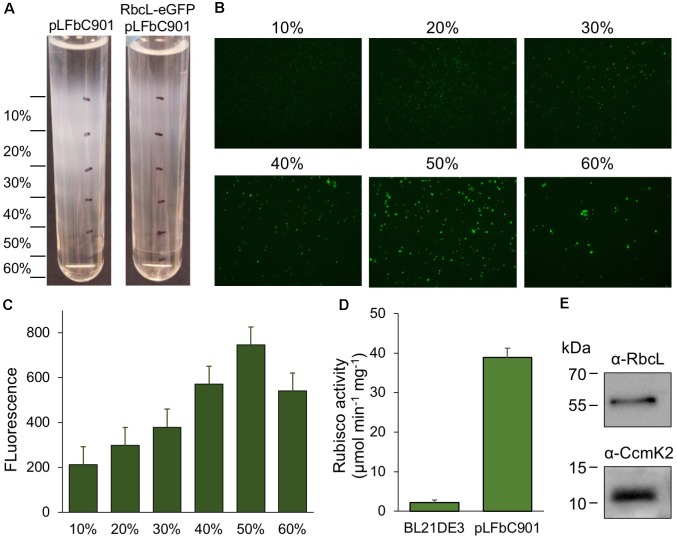
Purification and characterization of synthetic β-carboxysomes produced in *E. coli*. **(A)** Step sucrose gradient separation of synthetic β-carboxysomes with and without RbcL-eGFP-RbcS from pLFbC901. **(B)** Fluorescence detection of β-carboxysomes fused with eGFP in different sucrose fractions. **(C)** Fluorescence intensities of eGFP signals in different sucrose fractions, indicating the aggregation and abundance of β-carboxysome structures in each sucrose fraction. **(D)** Rubisco activities of β-carboxysomes in 50% sucrose fraction determined by ^14^C radiometric assay. **(E)** Immunoblot analysis of 50% sucrose fraction using α-RbcL and α-CcmK2 antibodies, corroborating the presence of interior (RbcL) and shell proteins (CcmK).

Purification of recombinant β-carboxysomes was performed by sucrose density gradient centrifugation, following the induction of *E. coli* constructs (**Figure 3A**). We used the RbcL-eGFP-RbcS and pLFbC901 co-expression strain to facilitate the detection of synthetic β-carboxysomes during purification. The eGFP tagging has been used in isolating natural β-carboxysomes from Syn7942 (Faulkner et al., 2017). The majority of synthetic β-carboxysome-like structures were detected in the 50% sucrose fractions by fluorescence imaging (**Figure 3B**). Rubisco assays of each β-carboxysome fraction also reveal that the 50% fraction presents the highest Rubisco activity (**Figure 3C**). By contrast, native β-carboxysomes from Syn7942 were found in the 40% fraction (Faulkner et al., 2017). This is ascribed to the relatively larger size of synthetic β-carboxysome-like structures (∼200 nm, **Figure 1B**) in contrast to native β-carboxysomes (∼150 nm) (Faulkner et al., 2017).

The same purification strategy was applied to isolate synthetic β-carboxysomes without eGFP fusion. Functional synthetic β-carboxysomes were also obtained in the 50% fraction, as confirmed by ^14^C radiometric Rubisco assays (**Figure 3D**). It is worth noting that the yield of synthetic β-carboxysomes was low and the carboxysome proteins were not clearly identifiable in SDS-PAGE (data not shown). However, immunoblot analysis using anti-RbcL and anti-CcmK2 revealed the presence of interior and shell proteins in isolated synthetic β-carboxysomes (**Figure 3E**).

**FIGURE 4:**
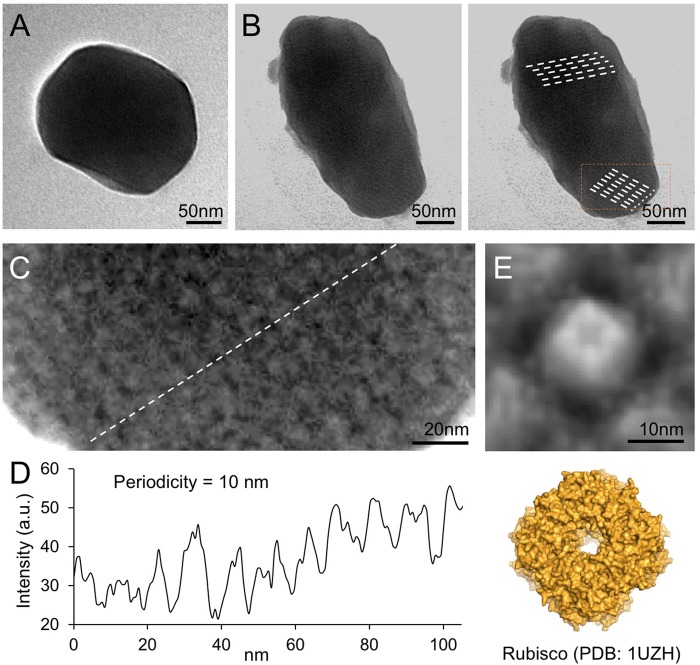
Negative-staining TEM images of isolated recombinant β-carboxysomes. **(A)** Representative β-carboxysome structures showing the polyhedral shape. The diameter of synthetic β-carboxysomes falls in the range of 200–300 nm. **(B)** Irregular β-carboxysome structures observed, implying the heterogeneity of recombinant β-carboxysomes. However, the densely packed paracrystalline arrays of interior proteins could be discerned (dash lines). **(C)** Zoom-in inspection of the paracrystalline arrays depicted in (**B**, rectangle). **(D)** Cross-section profile analysis of the paracrystalline arrays illustrates the periodic arrangement of interior proteins. The center-to-center distance between neighboring proteins is ∼10 nm. **(E)** Fourfold symmetrized correlation average TEM image of the interior protein (top) matches the atomic structure of Rubisco holoenzymes (bottom, PDB: 1UZH), indicating that the observed densely packed interior particles are Rubisco enzymes.

Electron microscopy images of purified β-carboxysomes describe that synthetic β-carboxysomes are 200–300 nm in diameter (**Figures 4A**,**B**), slightly larger and more heterologous than native β-carboxysomes from Syn7942 (Faulkner et al., 2017). Moreover, they do not exhibit manifestly a regular icosahedral shape and symmetry, though the straight edges and vertices are visible. Despite the possibility of being artifacts in sample preparation, the structural variations of native and synthetic β-carboxysomes may be a consequence of the change in protein abundance, ratio, and organization, which requires further optimization.

Closer inspection of the electron micrographs reveals the highly ordered paracrystalline arrays of interior proteins within the β-carboxysomal lumen (**Figures 4B,C**). Cross-section analysis illustrates that the center-to-center distance of the interior proteins is about 10 nm (**Figure 4D**). The specific protein arrangement observed in synthetic β-carboxysome-like particles is in good agreement with the Rubisco packing of the native β-carboxysome discriminated in previous studies (Kaneko et al., 2006; Faulkner et al., 2017). The individual proteins in the paracrystalline arrays depicted in the EM image were further analyzed using fourfold symmetrized cross-correlation single-particle averaging (Fechner et al., 2009; Liu et al., 2011b; Casella et al., 2017). The average protein structure matches well the atomic structure of Rubisco holoenzyme (**Figure 4E**). All of these results suggest that these proteins are Rubisco enzymes and are densely packed in the carboxysome-like structures.

**FIGURE 5:**
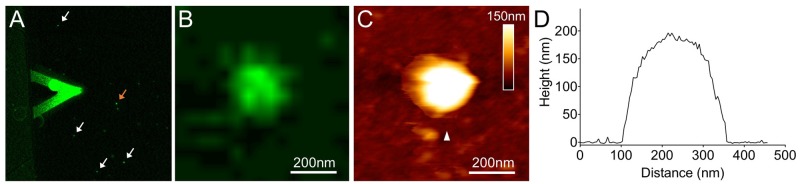
Combined confocal and AFM imaging of isolated synthetic β-carboxysomes fused with eGFP. **(A)** Merged microscopic image captured using a hybrid JPK AFM-Zeiss 880 confocal microscope. The triangular AFM cantilever is visible, and arrows indicate individual β-carboxysome particles immobilized on mica surface. **(B)** Fluorescence image of a single recombinant β-carboxysome indicated by the orange arrow in **(A)**. **(C)** AFM topograph of the β-carboxysome captured simultaneously with the fluorescence image **(B)**. The combination of AFM-confocal fluorescence imaging ensures the identification of intact recombinant β-carboxysomes. **(D)** Height profile of the β-carboxysome structure, taken along the white arrowhead indicated in **(B)**.

Furthermore, we studied the architecture of synthetic β-carboxysomes in solution using atomic force microscopy (AFM). The identification and structural integrity of eGFP-fused β-carboxysomes (RbcL-eGFP-RbcS) were verified by simultaneous AFM-fluorescence imaging (**Figure 5**). The single synthetic β-carboxysome is ∼250 nm large and ∼180 nm high, consistent with TEM results (**Figures 1B, 4A**).

### Expression of the Synthetic β-Carboxysome Operon in Syn7942

**FIGURE 6:**
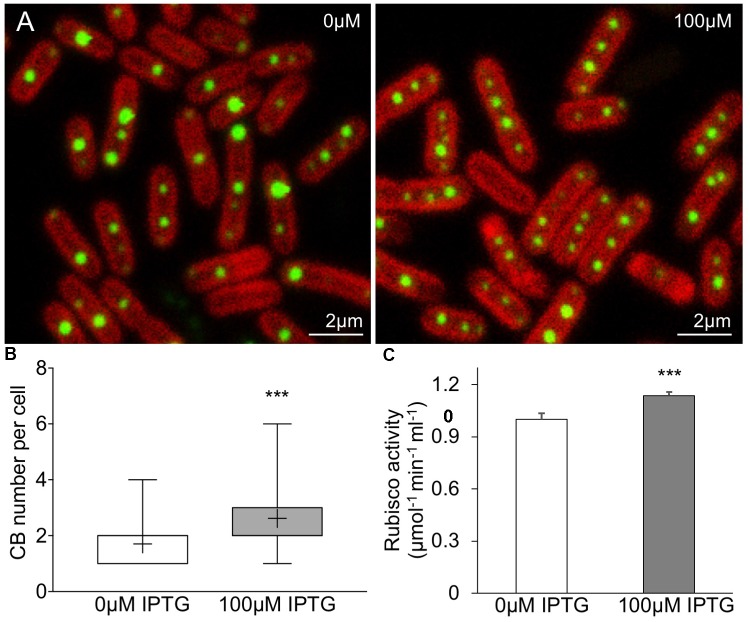
Enhancement of β-carboxysome formation in Syn7942 by the expression of pLFbC901. **(A)** Confocal microscopy images illustrating the β-carboxysome formation and abundance in Syn7942 cells induced by IPTG. Chlorophyll fluorescence is shown in red and fluorescently tagged β-carboxysomes are discerned as green puncta. **(B)** The number of β-carboxysomes per cell increases from 1.7 (no IPTG induction, *n* = 250) to 2.6 (100 μM IPTG induction, *n* = 350). The whiskers show data from minimum to maximum, “+” represents the average. Statistical analysis implies the significant difference (^∗∗∗^*p* < 0.001, two-tailed *t*-test). **(C)** *In vivo* ^14^C radiometric assays of Syn7942 cells induced by IPTG, illustrating a 13.7% increase in Rubisco activity resulted in 100 μM IPTG induction. Data are presented as mean ± SD. ^∗∗∗^*p* < 0.001 (*n* = 3, two-tailed *t*-test).

Our study shows that the designed synthetic carboxysome operon can produce functional β-carboxysomes in *E. coli*. To verify if the synthetic β-carboxysome operon can be expressed in Syn7942 cells, we inserted the synthetic β-carboxysome operon into a Syn7942 expression vector pAM2991 under the control of IPTG-inducible Ptrc promoter (Ivleva et al., 2005). The generated vector was then transformed into the RbcL-eGFP Syn7942 cells grown in low light (10 μE⋅m^-2^⋅s^-1^) and expressed in the presence of 100 μM IPTG (**Figure 6A**). The RbcL-eGFP Syn7942 cells express fluorescently tagged β-carboxysomes under endogenous genetic regulation (Sun et al., 2016). Confocal fluorescence images show that without IPTG induction, the Syn7942 cells harbor 1.7 ± 0.78 carboxysomes per cell (mean ± SD, *n* = 250, **Figure 6B**), consistent with the previous observation (Sun et al., 2016). IPTG induction results in an increase in carboxysome number per cell (2.6 ± 1.2, mean ± SD, *n* = 350). The carboxysomes are spatially distributed along the long axis of the cell. Moreover, Rubisco assays elucidate a 13.7% increase in carbon fixation activity per cell (**Figure 6C**). These results demonstrated that the expression of synthetic β-carboxysome operon triggered the formation of extra β-carboxysomes that were physiologically positioned and active in native hosts.

### Modularity and Interchangeability of the β-Carboxysome Structure

**FIGURE 7:**
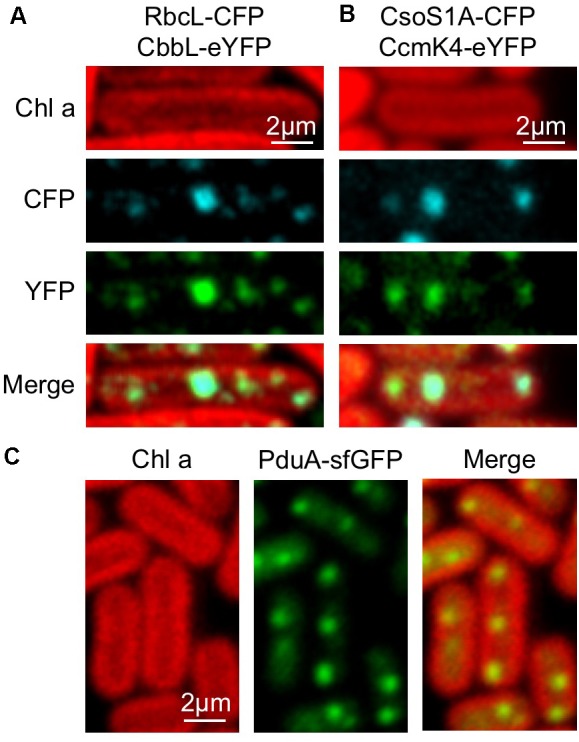
Interchangeability of Bacterial microcompartment (BMC) building blocks. **(A)** Co-localization of RbcL-CFP and CbbL-eYFP suggesting that α-carboxysome CbbL from *Halothiobacillus neapolitanus* can be encapsulated in the cyanobacterial β-carboxysome structure. **(B)** Co-localization of CsoS1A-CFP and CcmK4-eYFP implying the integration of α-carboxysome shell proteins from *H. neapolitanus* into the cyanobacterial β-carboxysome shell. **(C)** Evenly distributed fluorescence puncta of PduA-sfGFP along the longitude axis of Syn7942 cells indicating the incorporation of PDU shell proteins from *S. Typhimurium* in cyanobacterial β-carboxysomes.

The BMC shell is made of protein building blocks belonging to a family of homologous proteins (Yeates et al., 2011; Bobik et al., 2015). We evaluated the modularity of the β-carboxysome structure and interchangeability of building blocks from distinct types of BMCs. The Form 1A Rubisco large subunit CbbL of α-carboxysomes from *H. neapolitanus* was fused with eYFP at the C-terminus and expressed in RbcL-CFP Syn7942 cells. Confocal images reveal the co-localization of CbbL-eYFP and RbcL-CFP (**Figure 7A**), corroborating that α-carboxysome CbbL can be encapsulated in the β-carboxysome lumen that comprises Form IB Rubisco enzymes, given the high sequence similarity between CbbL and RbcL (78%, **Supplementary Figure 2**). Likewise, the α-carboxysome shell protein CsoS1A was fused with CFP at the C-terminus and expressed in CcmK4-eYFP Syn7942 cells. The notable co-localization of CsoS1A-CFP and CcmK4-eYFP elucidates the incorporation of α-carboxysome shell proteins into β-carboxysome structures (**Figure 7B**), consistent with the previous finding (Cai et al., 2015).

In addition, the PDU microcompartment shell protein PduA from *Salmonella enterica serovar Typhimurium* (*S. Typhimurium*) fused with superfolder GFP (sfGFP) was inserted in pAM2991 and expressed in Syn7942. PduA-sfGFP forms fluorescent puncta and present a typical distribution pattern of β-carboxysomes in the cytoplasm of Syn7942 cells, indicative of the integration of PDU BMCs shell proteins into the β-carboxysome structure (**Figure 7C**).

## Discussion

The self-assembly and modularity features and the specific role for CO_2_ fixation make carboxysomes a desirable engineering objective for metabolic enhancement and production of new nanomaterials. In this study, we show the construction of synthetic β-carboxysome operons and, to our knowledge, the first production of functional β-carboxysome-like structures in heterologous organisms. The structure and activity of the synthetic β-carboxysomes as well as the modularity of β-carboxysome structures were characterized using a combination of molecular genetics, Rubisco assays, proteomics and immunoblot analysis, confocal, electron, and AFM.

The synthetic β-carboxysome operon was created to assemble 12 β-carboxysome genes. The recombinant β-carboxysomes, generated by the expression of the synthetic β-carboxysome operon, harbor densely packed Rubisco enzymes, CcmM, and shell proteins (CcmK2, CcmK4), as confirmed by proteomic data (**Supplementary Table 2**), immunoblot analysis (**Figure 3E**), and Rubisco assays (**Figure 3D**). Our results reveal that these proteins are the main building blocks necessary for β-carboxysome formation and CO_2_ fixation. The “inside-out” model of β-carboxysome biogenesis suggests that the assembly of β-carboxysome is initiated from the Rubisco-CcmM aggregation into procarboxysomes (Cameron et al., 2013), followed by the addition of CcmN which has a C-terminal encapsulation peptide (Kinney et al., 2012) to recruit shell proteins and result in the encapsulation of CcmK2 and CcmO. Seven of the 12 β-carboxysome proteins were not detected, likely suggesting their low protein abundance in the β-carboxysome structure or the impeded protein expression (i.e., CcaA), probably due to the distant gene location from the T7 promoter. Nevertheless, the yield of synthetic β-carboxysomes after purification is low (data not shown), confirming the overall expression of β-carboxysome proteins in the recombinant *E. coli* is restricted. Whether there are negative effects of the production of carboxysome-like structures on the growth of *E. coli* cells remains unknown. Moreover, the sizes of synthetic and native β-carboxysomes are relatively comparable (**Figure 4**), whereas the shape of synthetic β-carboxysomes is not well defined as that of the natural form, likely as a result of improper content and ratio of expressed carboxysome proteins. All these observations highlight the necessity of optimizing the expression constructs and conditions in future work.

Despite the significance of Rubisco nucleation in shell encapsulation and as such β-carboxysome formation (Cameron et al., 2013), it has been demonstrated that synthetic β-carboxysome shell structures can be built by expressing the shell proteins CcmK1, CcmK2, CcmO, and CcmL (Cai et al., 2016). The synthetic β-carboxysome shells are about 25 nm in diameter, notably smaller than the native β-carboxysomes and synthetic β-carboxysomes produced in this study, suggesting the roles of interiors in determining the overall shape of β-carboxysomes.

The majority of cyanobacteria possess Form 1B Rubisco, the same as plants. However, compared with β-cyanobacteria Rubisco, plant Rubisco exhibits a relatively a low carboxylation activity but a high affinity for CO_2_ against O_2_. Improving the catalytic activity of plant Rubisco has become a target of plant engineering for enhanced photosynthesis and capacity (Carmo-Silva et al., 2015). One possible strategy is to engineer functional cyanobacterial carboxysomes into plants and encapsulate plant Rubisco with the carboxysome shell that has the selective permeability to HCO_3_^-^ and CO_2_. Prior to the present study, it has been demonstrated that expressing β-carboxysome shell proteins and cyanobacterial Form 1B Rubisco in tobacco chloroplasts is achievable (Lin et al., 2014a,b; Occhialini et al., 2016). Here, we describe the heterologous expression of functional β-carboxysomes in *E. coli*, which holds promise for plant chloroplast engineering to construct entire functional carboxysome structures for enhanced crop photosynthesis and productivity.

It is potentially challenging to perform multi-gene transformation in higher plants. The production of functional β-carboxysomes requires not only the expression of 12 carboxysome proteins and possibly other associated proteins, but also the manipulation of protein abundance and ratio. In a recent study, a chimeric protein peptide CcmC, including three Rubisco SSUs, one CcaA and the CcmN C-terminus encapsulation peptide, was designed to replace the endogenous β-carboxysome core (CcmM, CcmN, CcaA) (Gonzalez-Esquer et al., 2015). The reengineered β-carboxysomes were shown to support photosynthesis *in vivo*. In this study, we showed that the capacity of CO_2_ fixation can be obtained using a simplified carboxysome-expressing vector pLFbC601, which contains only seven β-carboxysome genes in the *ccmKLMNO* and *rbcLS* operons. This may represent an engineering objective to produce functional carboxysomes in higher plants with a simplified building composition. Optimization of the pLFbC601 expression to build functional CO_2_-fixing modules awaits further investigation.

Despite the functional diversity, all BMCs share a common structural feature: an icosahedral protein shell encapsulating internal catalytic enzymes (Bobik et al., 2015). The shell proteins have conserved sequences and form typical BMC domains: the PF00936 domain for hexameric shell proteins, the PF03319 domain for shell pentamers, and the PF00936 domain for trimeric shell proteins (Kerfeld et al., 2005; Tanaka et al., 2008; Klein et al., 2009). We demonstrate that the α-carboxysome shell protein CsoS1A from *H. neapolitanus*, the PDU shell hexameric protein PduA from *S. Typhimurium*, as well as the Form 1A Rubisco large subunit CbbL from *H. neapolitanus*, can be integrated into the native β-carboxysomes in Syn7942 cells, due to their sequence conservation and structural similarities relative to CcmK2 and RbcL, respectively. Our results shed lights on the inherent modularity and interchangeability of a range of BMC building blocks, which lay the foundation for generating chimeric BMC structures in a predictive manner, to manipulate the metabolic activities of BMCs in native and heterologous hosts and engineer BMC-based nanoreactors for specific functions. It will promote the “plug and play” applications of BMC-like structures in biotechnology.

## Materials and Methods

### Construction of Synthetic Operons, Plasmids, and Strains

The strategy for constructing synthetic β-carboxysome operons was illustrated in **Figure 1A**. Genes were cloned and synthesized from the genomic DNA of Syn7942 using the ligation-free method In-Fusion cloning (Clontech, Mountain View, CA, United States). Every operon contains 50 bp upstream and 20 bp downstream to preserve the native promoter and ribosome-binding site sequence of all the genes. The operons were inserted into either the pETM11 vector digested at the EcoRI and XhoI sites or the pAM2991 vector [a gift from Susan Golden, Addgene plasmid # 40248 (Ivleva et al., 2005)] at the EcoRI and BamHI sites. Genes encoding RbcL-eGFP-RbcS were cloned from the genomic DNA of the RbcL-eGFP Syn7942 mutant (Sun et al., 2016) and inserted into the pTTQ18 vector at the EcoRI and XbaI sites; the same strategy was used for cloning and inserting CcmK4-GFP at the EcoRI and KpnI sites. Genes encoding CsoS1A and CbbL were cloned from the genomic DNA of *H. neapolitanus* using In-Fusion cloning, fused with the genes of CFP and eYFP, respectively, and inserted into pAM2991. The *pduA* gene was cloned from the genome of *S. Typhimurium* LT2, fused with sfGFP, and inserted into pAM2991. Restriction enzymes were purchased from New England Biolabs (United Kingdom). Reagents were purchased from Sigma-Aldrich (United States).

Construction of plasmids and synthetic operons was carried out in *E. coli* strain Top10 or BL21(DE3) cells grown aerobically at 37°C in lysogeny broth (LB) medium. Medium supplements were used, where appropriate, at the following final concentrations: 50 μg⋅mL^-1^ kanamycin, 50 μg⋅mL^-1^ spectinomycin, and 100 μg⋅mL^-1^ ampicillin.

Syn7942 wildtype and RbcL-eGFP and CcmK4-eGFP mutants (Sun et al., 2016) were transformed with the generated pAM2991 vectors described above. Cells from single colonies were grown in BG11 media at a constant light until the middle of the exponential growth phase, and then induced with 100 μM IPTG overnight at 30°C.

### Expression of Synthetic β-Carboxysome Operons and Purification of Synthetic β-Carboxysomes

The transformed *E. coli* strain BL21DE3 was grown overnight at 37°C. The cultures were then diluted 1:100 in 1 L flasks of LB and incubated at 37°C with shaking until OD_600_ reaches 0.6, then induced with 50 μM IPTG and grow overnight at 18°C. Cells were harvested by centrifugation at 5,000 *g* for 10 min. Synthetic carboxysome purification was carried out as described previously Faulkner et al. (2017). The cell pellet was washed once with TE buffer, and was then resuspended into TE buffer in the presence of cell lytic B (Sigma-Aldrich, United States) and 1% protease inhibitor cocktails (Thermo Fisher, United Kingdom), and then cell breakage by Stansted Pressure Cell Homogenizer (Stansted Fluid Power, United Kingdom). Cell lysate was then treated with 1% *n*-dodecyl β-maltoside for 1 h. Cell debris was removed by centrifugation, followed by a centrifugation at 50,000 *g* to enrich synthetic β-carboxysomes. The generated pellet was resuspended in TE buffer and followed by centrifugation using a step sucrose density gradient. The sucrose fractions were then harvested and characterized to determine the presence and activities of carboxysome structures.

### SDS-PAGE, Immunoblot Analysis, Proteomic Analysis, and Rubisco Assay

SDS-PAGE, immunoblot analysis using anti-RbcL antibody were carried out as described previously (Sun et al., 2016; Faulkner et al., 2017). Immunoblot analysis of CcmK2 was performed using anti-CcmK2 antibody (a gift from Cheryl Kerfeld) and horseradish peroxidase-conjugated goat anti-rabbit immunoglobulin G secondary antibody (GE Healthcare, United States).

Whole-cell proteomic analysis was performed as follows: 20 mL of *E. coli* cells expressing synthetic β-carboxysome proteins were harvested. After washed and sonicated with 25 mM NH_4_HCO_3_, the cell lysates were used for mass spectrometry analysis, as described previously (Faulkner et al., 2017).

For *in vivo* Rubisco assays, 120 μL of *E. coli* cells (OD_600_ = 4.0) or 120 μL of Syn7942 cells (OD_750_ = 4.0) in Rubisco assay buffer (100 mM EPPS, pH 8.0; 20 mM MgCl_2_) were added into a 250 μL reaction. Radiometric assays were performed following the previously described protocol (Price and Badger, 1989) with additional cell permeabilization treatment (Schwarz et al., 1995). Rubisco activities of isolated synthetic β-carboxysome structures were determined as previously described Faulkner et al. (2017). Five microliters of 1 mg⋅mL^-1^ isolated synthetic β-carboxysomes in Rubisco assay buffer were added. Samples were added into scintillation vials containing NaH^14^CO_3_ at a final concentration of 25 mM and incubated at 30°C for 2 min before the addition of D-ribulose 1,5-bisphosphate sodium salt hydrate (RuBP, Sigma-Aldrich, United States) at a final concentration of 5 mM. The reaction ensued for 5 min before being terminated by adding 2:1 by volume 10% formic acid. Samples were dried for at least 30 min at 95°C to remove unfixed ^14^C before resuspending the fixed ^14^C pellets with ultra-pure water and adding 2 mL of scintillation cocktail (Ultima Gold XR, PerkinElmer, United States). Radioactivity measurements were then taken using a scintillation counter (Tri-Carb, PerkinElmer, United States). Raw readings were used to calculate the amount of fixed ^14^C, and then converted to the total carbon fixation rates. Results are presented as mean ± standard deviation (SD).

### Fluorescence Microscopy

*E. coli* BL21(DE3) cells possessing the *rbcL*-*egfp*-*rbcS*-pTTQ18 and *ccmK4*-pTTQ18 plasmids with or without pLFbC901 were cultured in 37°C until OD_600_ reaches 0.6 and then induced with 50 μM IPTG overnight at 18°C. Preparation of *E. coli* and Syn7942 cells for confocal microscopy was performed as described earlier (Liu et al., 2012). The *E. coli* and Syn7942 cells were imaged using a Zeiss LSM710 or LSM780 with a 63× or 100× oil-immersion objective and excitation at 488 nm. Live-cell images were recorded from at least five different cultures. All images were captured with all pixels below saturation. Image analysis was carried out using ImageJ software (NIH Image). Automated analysis of the number of carboxysomes in Syn7942 cells was programmed into the image analysis software ImageSXM^1^, as described earlier (Sun et al., 2016).

### Transmission Electron Microscopy

*Escherichia coli* cells expressing β-carboxysome proteins were pelleted and fixed for 1 h with 2% paraformaldehyde and 2% glutaraldehyde in 0.1 M sodium cacodylate buffer at pH 7.2. Samples were stained for 1 h with 2% osmium tetroxide and 1.5% potassium ferrocyanide, followed by staining with 1% thiocarbohydrazide for 20 min and 2% osmium tetroxide for 30 min. Afterwards, samples were washed with ultrapure water, stained with 1% uranyl acetate for overnight at 4°C. Dehydration was performed with a series of increasing alcohol concentrations (30 to 100%) before the samples were embedded in resin. Thin sections of 70 nm were cut with a diamond knife. The structures of isolated carboxysomes were characterized using negative staining TEM and AFM imaging as described previously (Faulkner et al., 2017). Samples were stained with 3% uranyl acetate. Images were recorded using an FEI Tecnai G2 Spirit BioTWIN transmission electron microscope. Image analysis was carried out using ImageJ software (NIH Image). Particle averaging was performed using cross-correlation based Java routines for the ImageJ image processing package (Fechner et al., 2009; Liu et al., 2011b; Casella et al., 2017).

### Atomic Force Microscopy

AFM imaging was carried out in solution to ensure the structural and functional integrity of β-carboxysomes (Faulkner et al., 2017). Confocal-AFM images were captured using a NanoWizard 3 AFM (JPK) integrated with a Zeiss LSM880 confocal microscope. Samples were adsorbed on glass slides in adsorption buffer (10 mM Tris–HCl, 150 mM KCl, 25 mM MgCl_2_, pH 7.5) for 10 min, and then washed with imaging buffer (10 mM Tris–HCl, 150 mM KCl, pH 7.5) (Liu et al., 2009, 2011a,b; Casella et al., 2017). Confocal images were captured using a 40× objective with 488 nm excitation.

Particles with high-intensity GFP signal were imaged by AFM in Quantitative Imaging (QI) mode. The scanning force is ∼100 pN. Image analysis was performed using JPK SPM Data Processing (JPK).

## Author Contributions

YF and L-NL conceived the experiments. YF, FH, MF, QJ, GD, and MY conducted the experiments. YF, FH, MF, and L-NL analyzed the results. YF and L-NL wrote the manuscript. All authors reviewed the manuscript.

## Conflict of Interest Statement

The authors declare that the research was conducted in the absence of any commercial or financial relationships that could be construed as a potential conflict of interest.

## Footnotes

1 http://www.imagesxm.org.uk/
