# Supplementary material for: Engineering and Modulating Functional Cyanobacterial CO2-Fixing Organelles
Source: Front Plant Sci. 2018 Jun 5;9:739. doi: 10.3389/fpls.2018.00739 (PMC5996877; doi:10.3389/fpls.2018.00739)
Supplement: Supplementary file 1 [file Presentation_1.pdf]

Supplementary Material

Engineering and modulating functional cyanobacterial CO_2-fixing organelles

Yi Fang[1], Fang Huang[1], Matthew Faulkner[1], Qiuyao Jiang[1], Gregory F. Dykes[1], Mengru Yang[1], and Lu-Ning Liu[1*]

[1]Institute of Integrative Biology, University of Liverpool, Crown Street, Liverpool L69 7ZB, United Kingdom

*** Correspondence:** Dr Lu-Ning Liu: luning.liu@liverpool.ac.uk

Supplementary Figures and Tables

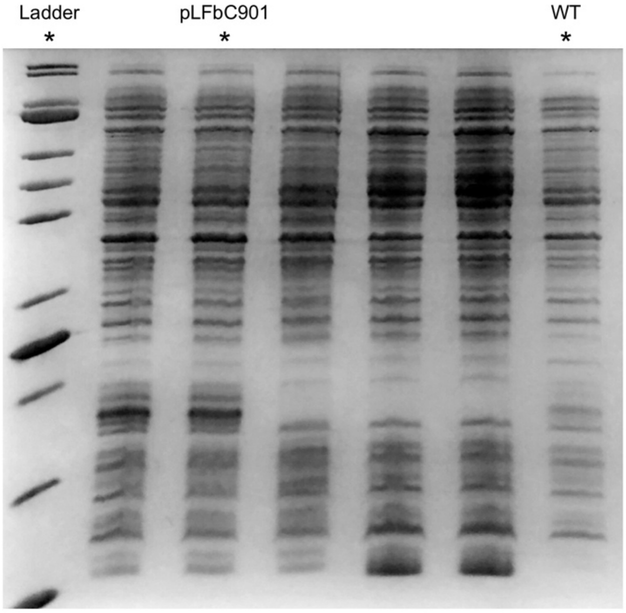

Supplementary Figure 1. Raw SDS-PAGE gel image of the total cell extracts of the recombinant *E. coli* pLFbC901 and the *E. coli* WT cells. The sample lanes of protein ladder, WT and pLFbC901 were selected to be shown in Figure 1C.

A

```
CbbL        ----MAVKKYSAGVKEYRQTYWMPEYTPLDSDILACFKITPQPGVDREEAAAAVAAESST
RbcL        MPKTQSAAGYKAGVKDYKLTYYTPDYTPKDTDLLAAFRFSPQPGVPADEAGAAIAAESST
                 :.  *.****:*: **: *:*** *:*:**.*:::*****  :**.**:******

CbbL        GTWTTVWTDLLTDMDYYKGRAYRIEDVPGDDAAFYAFIAYPIDLFEEGSVVNVFTSLVGN
RbcL        GTWTTVWTDLLTDMDRYKGKCYHIEPVQGEENSYFAFIAYPLDLFEEGSVTNILTSIVGN
            *************** ***:.*:** * *:: :::******:********.*::**:***

CbbL        VFGFKAVRGLRLEDVRFPLAYVKTCGGPPHGIQVERDKMNKYGRPLLGCTIKPKLGLSAK
RbcL        VFGFKAIRSLRLEDIRFPVALVKTFQGPPHGIQVERDLLNKYGRPMLGCTIKPKLGLSAK
            ******:*.*****:***:* ***  *********** :******:**************

CbbL        NYGRAVYECLRGGLDFTKDDENINSQPFMRWRDRFLFVQDATETAEAQTGERKGHYLNVT
RbcL        NYGRAVYECLRGGLDFTKDDENINSQPFQRWRDRFLFVADAIHKSQAETGEIKGHYLNVT
            **************************** ********* ** ..::*:*** ********

CbbL        APTPEEMYKRAEFAKEIGAPIIMHDYITGGFTANTGLAKWCQDNGVLLHIHRAMHAVIDR
RbcL        APTCEEMMKRAEFAKELGMPIIMHDFLTAGFTANTTLAKWCRDNGVLLHIHRAMHAVIDR
            *** *** ********:* ******::*.****** *****:******************

CbbL        NPNHGIHFRVLTKILRLSGGDHLHTGTVVGKLEGDRASTLGWIDLLRESFIPEDRSRGIF
RbcL        QRNHGIHFRVLAKCLRLSGGDHLHSGTVVGKLEGDKASTLGFVDLMREDHIEADRSRGVF
            : *********:* **********:**********:*****::**:**..*  *****:*

CbbL        FDQDWGSMPGVFAVASGGIHVWHMPALVNIFGDDSVLQFGGGTLGHPWGNAAGAAANRVA
RbcL        FTQDWASMPGVLPVASGGIHVWHMPALVEIFGDDSVLQFGGGTLGHPWGNAPGATANRVA
            * ***.*****:.***************:**********************.**:*****

CbbL        LEACVEARNQGRDIEKEGKEILTAAAQHSPELKIAMETWKEIKFEFDTVDKLDTQNR
RbcL        LEACVQARNEGRDLYREGGDILREAGKWSPELAAALDLWKEIKFEFETMDKL-----
            *****:***:***: :** :**  *.: ****  *:: ********:*:***
```

B

```
CcmK2       ----MPIAVGMIETLGFPAVVEAADAMVKAARVTLVGYEKIGSGRVTVIVRGDVSEVQAS
CsoS1A      MADVTGIALGMIETRGLVPAIEAADAMTKAAEVRLVGRQFVGGGYVTVLVRGETGAVNAA
PduA        ---MQQEALGMVETKGLTAAIEAADAMVKSANVMLVGYEKIGSGLVTVIVRGDVGAVKAA
                   *:**:** *:  .:******.*:*.* *** : :*.* ***:***:.. *:*:

CcmK2       VSAGLDSAKRVAGGEVLSHHIIARPHENLEYVLPIRYTEAVEQFRM
CsoS1A      VRAGADACERVGDG-LVAAHIIARVHSEVENILPKAPQA-------
PduA        TDAGAAAARN--VGEVKAVHVIPRPHTDVEKILPKGISQ-------
            . **  :...   * : : *:* * * ::* :**
```

Supplementary Figure 2. Sequence alignment of representative BMC proteins using Clustal Omega multiple sequence alignment program. (A) Sequence similarity (78%) between the Form 1B Rubisco large subunit CbbL from *H. neapolitanus* and the Form 1B Rubisco large subunit RbcL from Syn7942. (B) Sequence conservation of representative BMC shell hexamers, CcmK2 from Syn7942, CsoS1A from *H. neapolitanus* and PduA from *S. Typhimurium* (CsoS1A – CcmK2: 53%; PduA – CcmK2: 61%).

Primers	Sequences	Description
Long fragment F<br>Long fragment R	CCCCTGCCAGTCTCTTCTGATTAAGGTTTGCTTCTTGCTAGAGAC<br>TCAACATATTCTACTATCAAGACAAATCAGGCTTTAG	For pLFbC901-pETM11
Short fragment F<br>Short fragment R	TTTGTCTTGATAGTAGAATATGTTGATGGCTCTTC<br>ATCTCAGTGGTGGTGGTGGTGGTGCAATCAACGTGTTGAACAATTTC	
6k F<br>6k R	CCCCTGCCAGTCTCTTCTGATTAAG GTTTGCTTCTTGCTAGAGAC<br>ATCTCAGTGGTGGTGGTGGTGGTGCCTATCAAGACAAATCAGGCTTTAG	For pLFbC601-pETM11
RbcL F<br>RbcL R	ACAGGAAACAGCGATGCCCAAGACGCAATCTGCC<br>GCCTGCAGGTCGACTTTAGTAGCGGCCGGGACG	For *rbcL-egfp-rbcS*-pTTQ18
ccmK4 F<br>ccmK4 R	GCGCGAATTCGATGTCTCAGCAGGCAATTGG<br>GCGCGGTACCATTCCGGGGATCCGTCGACC	For *ccmK4-egfp*-pTTQ18
b-cb-pAM2991 F<br>b-cb-pAM2991 R	AGGAAACAGACCATGGGTTTGCTTCTTGCTAGAG<br>CAGGTCGACTCTAGAGAATCAACGTGTTGAACAATTTC	For pLFbC901-pAM2991 express in Syn7942
CsoS1A F<br>CsoS1A R	AGGAAACAGACCATGGAGGCTTGTTCCGGTTCTTTG<br>CTCCGGGCCCGGCAGTTAGGCTTGTGGCGCCTT	For CsoS1A-CFP- pAM2991 express in Syn7942
CFP F<br>CFP R	CTGCCGGGCCCGGAGCTG<br>CAGGTCGACTCTAGAG TTAAGCGCTTACGTAGAGC	
CbbL F<br>CbbL R	AGGAAACAGACCATGGATGGCAGTTAAAAAGTATAGTG<br>CTCCGGGCCCGGCAGTCAACGATTTTGAGTGTCGA	For CbbL-YFP-pAM2991 express in Syn7942
YFP F<br>YFP R	CTGCCGGGCCCGGAGCTG<br>CAGGTCGACTCTAGAGTTACTTGTACAGCTCGTC	
pduA F<br>pduA R	ACACAGGAAACAGACCATGGCCCAACTATCGGAACACTC<br>GGAGCCAGCGGATCCTTGGCTAATTCCCTTCGGTA	For PduA-sfGFP-pAM2991 express in Syn7942
sfGFP F<br>sfGFP R	GCCAAGGATCCGCTGGCTCCGCTGC<br>CCTGCAGGTCGACTCTAGAGAAGGAGGATATTCATATGTTATTTG	

Supplementary Table 1. Oligonucleotides used in cloning.

CyanoBase	UniProt	Protein Description	Masco t score	Coverage (%)	Unique peptides
Synpcc7942_1426	Q31NB3	Rubisco large chain RbcL	206	14	KLTYYTPDYTPKD<br>KDTDLLAAFRF<br>RAVYECLRG<br>KASTLGFVDLMRE<br>REDHIEADRS<br>KWSPELAAALDLWKE
Synpcc7942_1427	Q31NB2	Rubisco small chain RbcS	178	42	RFETFSYLPPLSDRQ<br>KLPLFDCKS<br>KSPQQVLDEVRE<br>RSEYGDCYIRV<br>RVAGFDNIKQ
Synpcc7942_1421	Q03511	CcmK2	155	40	RVTLVGYEKI<br>RVTVIVRG<br>RGDVSEVQASVSAGLDSAKR<br>RYTEAVEQFRM
Synpcc7942_1423	Q03513	CcmM	129	6	RLSSEVITQVRS<br>RNLLAQGYRI<br>RVFEALIQDPQGPVGSAKA
Synpcc7942_0285	Q31RK2	CcmK4	112	24	RFAVNIRG<br>KTAMDAGIEAAKN<br>RLSAEGTGSGRR

Supplementary Table 2. Proteomic analysis of identified β-carboxysome proteins expressed by pLFbC901 in the whole *E. coli* cell extract.
